# Phosphorylation of the IDP KID Modulates Affinity for KIX by Increasing the Lifetime of the Complex

**DOI:** 10.1016/j.bpj.2017.10.015

**Published:** 2017-12-19

**Authors:** Liza Dahal, Sarah L. Shammas, Jane Clarke

**Affiliations:** 1 Department of Chemistry, University of Cambridge, Cambridge, United Kingdom

## Abstract

Intrinsically disordered proteins (IDPs) are known to undergo a range of posttranslational modifications, but by what mechanism do such modifications affect the binding of an IDP to its partner protein? We investigate this question using one such IDP, the kinase inducible domain (KID) of the transcription factor CREB, which interacts with the KIX domain of CREB-binding protein upon phosphorylation. As with many other IDPs, KID undergoes coupled folding and binding to form *α*-helical structure upon interacting with KIX. This single site phosphorylation plays an important role in the control of transcriptional activation in vivo. Here we show that, contrary to expectation, phosphorylation has no effect on association rates—unphosphorylated KID binds just as rapidly as pKID, the phosphorylated form—but rather, acts by increasing the lifetime of the complex. We propose that by controlling the lifetime of the bound complex of pKID:KIX via altering the dissociation rate, phosphorylation can facilitate effective control of transcription regulation.

## Introduction

Disordered proteins are highly abundant in eukaryotic cells and are involved in many key biological functions, including cell signaling and regulation of transcription (1, 2, 3). Their flexible and dynamic structures make them susceptible to posttranslational modifications (PTMs), such as phosphorylation, methylation, and SUMOylation, which can facilitate remarkable functional diversity (4, 5, 6). In living cells, PTMs allow intrinsically disordered proteins (IDPs) to trigger different cellular responses by controlling their interactions with partner proteins (7, 8).

Phosphorylation is the most commonly studied PTM (9, 10); almost 2% of human protein-coding genes encode for protein kinases, highlighting the importance of phosphorylation as a regulatory mechanism (11). IDPs that are involved in cell signaling and regulation are enriched with phosphorylation sites (12). In IDPs, phosphorylation has been shown to induce conformational changes (13, 14, 15, 16) and also to activate or deactivate cellular signals by promoting order-disorder transitions (17, 18, 19). Addition of negatively charged phosphate groups may also make long-range electrostatic contributions to binding affinity (20, 21): in some IDPs with multiple phosphorylation sites, several phosphate groups are known to change bulk electrostatics and can work cooperatively as ultrasensitive rheostats (21, 22, 23, 24).

**Figure 1:**
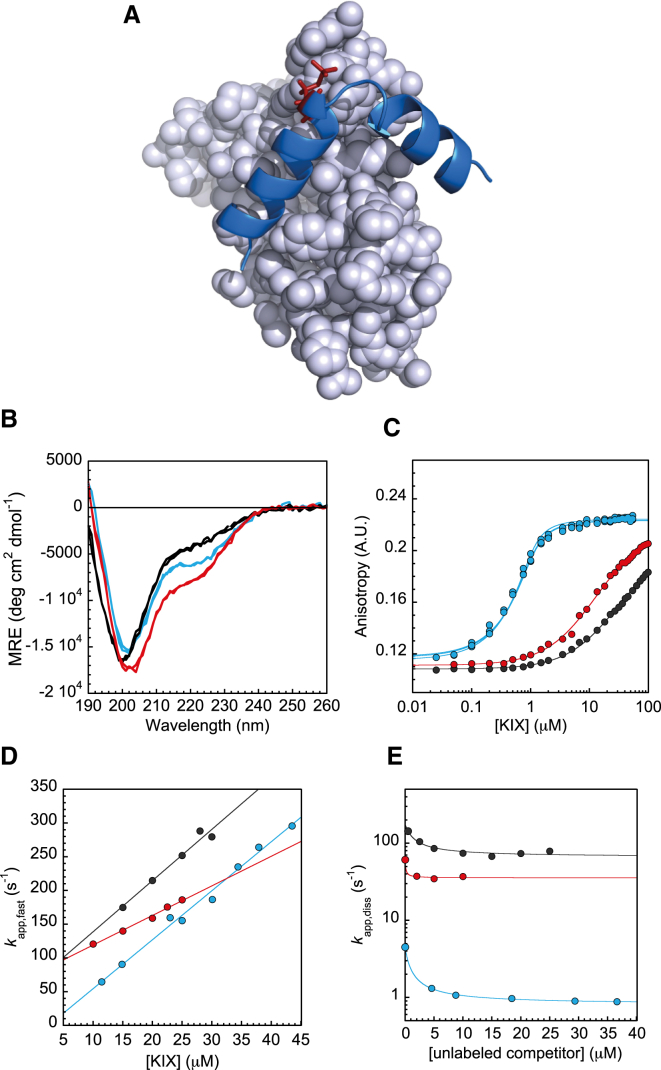
(*A*) Interactions of KID with KIX. When disordered pKID (*blue*) binds to KIX (*gray*) it forms two helices with a kinked region between the helices. The phosphorylated Ser 133 is shown in red. (*B*) CD spectra. Unphosphorylated KID (*black*) is significantly disordered. Phosphorylation increases the amount of residual helicity in pKID (*blue*), as does the S133E mutation in KID-S133E (*red*). (*C*) Equilibrium measurements. pKID binds to KIX significantly more tightly than KID or KID-S133E (colors as in *B*). (*D*) Association kinetics (fast rates only shown). The gradient gives the association rate constant. KID and pKID have the same rate constant, but KID-S133E binds more slowly (colors as in *B*). (*E*) Dissociation kinetics. pKID dissociates from the complex with KIX significantly more slowly than KID or KID-S133E (colors as in *B*).

The intrinsically disordered kinase inducible domain (KID) of cyclic-AMP response element binding protein (CREB) contains several phosphorylation sites, but phosphorylation of just a single serine residue is able to modulate signaling (25, 26, 27). Protein kinase A phosphorylates S133 in the KID domain of CREB, thereby increasing its binding affinity for the KIX domain of the coactivator CREB-binding protein (28, 29, 30). The interaction between phosphorylated KID (pKID) and KIX is a coupled folding and binding reaction, resulting in the pKID domain folding into a kinked helical structure upon binding to KIX (Fig. 1 *A*) (29, 31). In-cell studies show that interaction of pKID with KIX triggers recruitment of the transcription machinery to CREB-response element sites, which then modulates transcription of genes important for, among other things, circadian rhythm and long-term memory (32, 33).

Here, we investigate how phosphorylation increases the affinity of KID for KIX. Contrary to our expectation, phosphorylation has no effect on the binding rate constant, despite affecting both the charge and residual structure of KID. Instead, the binding affinity is increased due to a change in the lifetime of the bound complex.

## Materials and Methods

### Expression and purification of KIX and pKID

Expression and purification of KIX was carried out as described previously (34). pKID for labeling with external Alexa dyes was expressed using the coexpression vector and purification was carried out as outlined in Sugase et al. (35). A single cysteine mutation was introduced at the N-terminus of pKID (sequence shown in Supporting Material) using site-directed mutagenesis. Subsequently, labeling with Alexa 488, Alexa 546 and Alexa 594 C5 maleimide (Molecular Probes, OR, Eugene; Life Technologies, Carlsbad, CA) was carried out as described previously (36).

### Peptides

Fluorescein isothiocyanate (FITC)-KID (UniProt P15337, residues 116-146, N-terminus labeled with FITC), FITC-pKID, and FITC-KID-S133E were purchased from Biomatik (Ontario, Canada). As required, 2.0 mg aliquots were dissolved at 2 mg mL^−1^ with 100 mM sodium phosphate buffer (pH 7.5). The concentration of IDP was determined from absorbance at 495 nm of a 40-fold dilution, using the extinction coefficient 58600 M^−1^cm^−1^. This extinction coefficient was calculated by comparison with concentration determinations from amino acid analysis of multiple peptide samples.

The concentrations of Alexa-labeled pKID were determined from absorbance at 495, 556, and 590 nm using extinction coefficients 72,000, 104,000, and 73,000 M^−1^cm^−1^ for Alexa 488, Alexa 546, and Alexa 594, respectively. The concentration of Y-pKID (unlabeled pKID) was determined using absorbance of tyrosine (Tyr) at 280 nm using the extinction coefficient 1474 M^−1^cm^−1^. All calculated concentrations for unlabeled pKID and Alexa-labeled pKID were then corrected using amino acid analysis of peptide samples.

### Biophysical buffers and dilutions

100 mM sodium phosphate (pH 7.5) was used for all experiments. 0.05% Tween was included in all dilutions to avoid problems with peptides adhering to plastic surfaces. When preparing solutions for kinetic studies, all dilutions were weighed to achieve accurate concentrations.

### Circular Dichroism

Samples were prepared at different concentrations using 100 mM sodium phosphate (pH 7.5) and 0.05% Tween-20. Circular dichroism (CD) spectra (190–260 nm) were recorded in a 1 mm pathlength cuvette using a ChiraScan CD Spectrometer from Applied Photophysics. All spectra are presented with buffer subtracted. Predicted helicities were calculated using the methods described previously (37, 38).

### Fluorescence anisotropy equilibrium binding curves

FITC-pKID, FITC-KID, and FITC-KID-S133E (1 *μ*M) samples were incubated at 10°C for 30 min in the presence of varying concentrations of KIX. Measurements were performed using a Cary Eclipse Spectrophotometer with a fluorescence polarization accessory. Excitation and emission wavelengths of 495 ± 5 and 515 ± 5 nm, respectively, were used. The sample holder was maintained at 10°C with a Peltier device. Fluorescence anisotropy equilibrium binding experiments and calculations were performed as described previously (34).

### Kinetic measurements

Kinetic experiments were performed using a SX18 fluorescence stopped-flow spectrometer from Applied Photophysics. The temperature was maintained at 10°C. Briefly, association experiments were performed at 10-fold (or larger) excess of KIX over FITC-KID, FITC-pKID, and FITC-KID-S133E, and also 10-fold (or larger) excess of FITC-pKID over KIX, to generate pseudo-first-order conditions. The traces were fit to an equation describing a double exponential process, as two phases were observed. The dependence of the observed fast association rates on [KIX] was fit to a straight line where the gradient represents the fast association rate constant (*k*_ass,fast_). Out-competition dissociation experiments were carried out by rapid mixing of KIX:FITC-(p)KID with an unlabeled competitor peptide (cMybTAD, the transactivation domain of cMyb that binds to KIX at the same site as pKID). All kinetic traces were fit to single exponentials and the observed apparent dissociation rate constants were plotted as a function of different concentrations of unlabeled cMybTAD. The data were fit to Eq. 1 as discussed in Shammas et al. (36). The asymptote of the fit in this case represents the dissociation rate constant (*k*_diss_),

(1) $$k_{\text{obs}}^{\text{off}}=k_{\text{diss}}+\;k_{\text{on}}\left[\text{KIX}\right]\frac{1}{1+\frac{\left[\text{unlab}\right]}{K_{{\text{d}}_{\text{unlab}}}}},$$

where [unlab] is the concentration of unlabeled competitor used to displace labeled pKID, and *K*_dunlab_ is the equilibrium dissociation constant between the unlabeled peptide and KIX.

## Results

### Biophysical characterization of the binding of pKID

Previous NMR studies have suggested that the folding and binding of pKID to KIX is a three-state reaction (31). However, there are to date no stopped-flow kinetic investigations of the kinetics of association and dissociation. Thus, we needed first to establish conditions where this could be investigated. The binding of pKID to KIX results in a change in Tyr fluorescence, but only a single kinetic phase is observed (Fig. S1; Table S1). This, we reasoned, was either because the binding of pKID to KIX is essentially two-state under the conditions of our study or because the change in Tyr fluorescence does not report both kinetic phases. We tested a number of pKID variants with extrinsic fluorescent dyes attached to the N-terminus (Table S1). In the case of Alexa 546 and FITC, two kinetic phases could be detected (Table S1). Only a single phase was observed in the case of Alexa 488 and Alexa 594, although we note that the overall amplitude was extremely low in the case of Alexa 594 (Table S1). Where dyes were used, the apparent association rate increased by up to 4-fold, compared with that determined using Tyr fluorescence on the unlabeled peptide (determined at a single concentration of KIX, 31 *μ*M) (Table S1). As labeling efficiencies for the Alexa dyes were low, we decided to use synthesized FITC-KID and FITC-pKID (N-terminal labeled with FITC) for all the biophysical experiments reported below.

### The effect of phosphorylation on equilibrium binding affinity

To investigate the role of phosphorylation in the KID-KIX interaction, we examined the behavior of unphosphorylated FITC-KID and phosphorylated FITC-pKID. CD spectra revealed that FITC-KID and FITC-pKID are both mainly disordered under our experimental conditions (Fig. 1 *B*); the overall helicity was calculated to be around 11 and 17%, respectively (5, 37, 38). Thus, as has been observed previously, phosphorylation slightly increases the overall helicity of the peptide (30, 39, 40, 41, 42). We also observed the expected enhancement of KIX binding by phosphorylation of FITC-KID (29, 32, 41). The dissociation constant (*K*_d_) for FITC-pKID with KIX, under our experimental conditions, was determined by fluorescence anisotropy to be 0.11 ± 0.02 *μ*M (SD *n* = 3) (Fig. 1 *C*; Table 1). The affinity of FITC-KID is two orders of magnitude lower: the *K*_d_ for FITC-KID was estimated to be 29 ± 1 *μ*M (Fig. 1 *C*; Table 1).

**Table 1:** Experimental Results for KID, pKID, and KID-S133E

FITC-Peptide	*k*_ass,fast_ (*μ*M^−1^s^−1^)	*k*_ass,slow_ (s^−1^)	*K*_d_ (*μ*M)	*k*_diss_ (s^−1^)
KID	7.6 ± 0.8	21.2 ± 2.4	29.0 ± 1.0	66.3 ± 4.9
pKID	7.3 ± 0.3	24.2 ± 1.4	0.11 ± 0.02	0.81 ± 0.01
KID-S133E	4.4 ± 0.2	17.0 ± 1.0	12.3 ± 0.5	35.6 ± 2.2

### Association kinetics of FITC-pKID with KIX

In association experiments performed under pseudo-first-order conditions (with KIX in >10-fold excess over FITC-pKID) two phases were observed. Under our experimental concentration range the faster of the two rates appeared linearly dependent upon concentration (*k*_ass,fast_ = 7.3 ± 0.3 *μ*M^−1^ s^−1^). The second (slower) rate was hard to determine accurately due to low amplitude, but displayed little or no concentration dependence and is around 24 s^−1^ (Fig. S2; Table 1). We examined the behavior under a similar concentration range but with FITC-pKID in excess over KIX (ratio 1:10). The observed rates were essentially the same under these “reversed” pseudo-first-order conditions; we still observe a fast, linear, concentration-dependent rate (*k*_ass,fast_ = 6.9 ± 0.8 *μ*M^−1^ s^−1^) and a second slower rate (*k*_ass,slow_ ∼20 s^−1^) (Fig. S2).

### The effect of dephosphorylation on association and dissociation kinetics

In association kinetics experiments two phases are still observed for unphosphorylated FITC-KID. Interestingly, both rates are about the same as for FITC-pKID (*k*_ass,fast_ = 7.6 ± 0.8 *μ*M^−1^ s^−1^, *k*_ass,slow_ ∼21 s^−1^) (Figs. 1 *D* and S3; Table 1).

**Figure 2:**
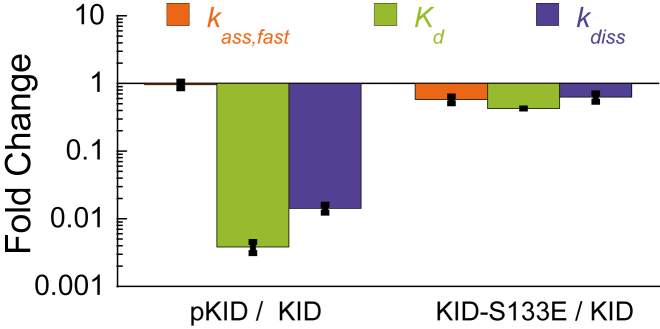
Comparison of the effects of phosphorylation and mutation to Glu of residue Ser 133 on binding kinetics and affinity. *k*_ass,fast_ is represented in orange, *K*_d_ in green, and *k*_diss_ in purple.

In contrast, dissociation experiments performed by out-competing with unlabeled cMybTAD display only a single phase. The dissociation rate constant (*k*_diss_) for phosphorylated FITC-pKID was determined to be 0.81 ± 0.01 s^−1^ (Fig. 1 *E*; Table 1). For FITC-KID, *k*_diss_ increases significantly to 66.3 ± 4.9 s^−1^, ∼80-fold faster than FITC-pKID (Figs. 1 *E* and 2; Table 1).

### Phosphomimetics do not recapitulate pKID

Glutamic acid is often used as an alternative for phosphate when studying phosphorylated peptides (43, 44, 45). Parker et al. (46) have previously shown glutamate cannot substitute for phosphorylated Ser in KID-KIX binding assays, but there was no mechanistic explanation. To understand the effect of the Ser-to-Glu substitution in FITC-KID on binding kinetics, we investigated a variant, FITC-KID-S133E, where the serine residue, which undergoes phosphorylation in KID, is substituted with glutamic acid.

As with phosphorylation, substitution of S133 by Glu increases the helicity of KID (Fig. 1 *B*). The overall residual helicity was calculated to be 22%. We observed a roughly 2-fold increased affinity of KIX binding to the phosphomimetic in comparison to FITC-KID (*K*_d_ = 12.3 ± 0.5 *μ*M) (Fig. 1 *C*; Table 1). The equilibrium binding affinity is still approximately two orders of magnitude lower than FITC-pKID (Fig. 2). Dissociation of FITC-KID-S133E is some two-times slower than for FITC-KID (*k*_diss_ = 35.6 ± 2.2 s^−1^) and ∼40-fold faster than FITC-pKID (Figs. 1 *E* and 2; Table 1). To our surprise, association is also slightly slower; the fast rate (*k*_ass,fast_) decreases to 4.4 ± 0.2 *μ*M^−1^s^−1^ (Fig. 1 *D*; Table 1), although the slow rate is similar to that of FITC-pKID and FITC-KID (Fig. S3; Table 1).

## Discussion

### Association is through an induced fit mechanism

Although IDP association/dissociation reactions are often apparently two-state, in principle there must be at least two steps in both association and dissociation, no matter what the mechanism (47). Two extreme mechanisms of binding are proposed: the IDP (pKID) either binds to its partner first and then folds (induced fit), or it folds to a binding competent conformation first, which then binds (conformational selection) (47, 48). Of course it is likely that both mechanisms may operate at one time, or the mechanisms may involve aspects of both; for example, a partly structured conformation of IDP binds to the partner before folding further, to form a fully bound complex.

It has been shown that the mechanisms can be discriminated by reversing the pseudo-first-order conditions (47, 48, 49). Here, our reversed experiments using pKID in excess (ratio 1:10) reveal that the observed rates are the same as those observed with KIX in excess (Fig. S2). This is consistent with an induced fit mechanism as has been suggested previously based both on NMR measurements and on the observation that the association of pKID with KIX is very fast (31, 36, 42, 50). Note that the mechanism is probed in more detail in the accompanying article by Dahal et al. in this issue of *Biophysical Journal* (51).

### Comparison with NMR kinetics

Comparing studies performed on different constructs under different conditions can be problematic. As an example: Wright and co-workers have reported the *K*_d_ for the pKID:KIX interaction to be dependent on length of the construct. Where the same entire binding region was included, then extension beyond the binding site stabilized the complex by up to ∼4-fold (pKID^29^ [residues 119–147], pKID^34^ [residues 116–149], and pKID^60^ [residues 101–160] had *K*_d_ values of 3.1, 1.3, and 0.7 *μ*M, respectively) (39, 52). This may not result from specific interactions: similar effects on complex stability have been shown to be due to alterations in overall electrostatics, with charge contributions from flanking residues (36). Furthermore, the experimental conditions (e.g.*,* salt, pH, and temperature) can also be expected to affect binding affinity (e.g., lowering the pH from 7.0 to 5.5 has been shown to raise the *K*_d_ for pKID^29^ by >2-fold, to 8.3 *μ*M) (52).

Thus it is difficult to directly compare the kinetic study we have performed here with that from the NMR experiments of Sugase et al. (31). In what follows it must be noted that we are not comparing like with like. In the previous study the experiments were performed on pKID^32^ (116–147) at pH 7.0 (20 mM Tris-d_11_-acetate-d_4_), at 30°C, at relatively low ionic strength (50 mM NaCl added to give an ionic strength *I*, of 69 mM). In our study, the pKID construct was one residue shorter (31 residues, 116–146) and labeled with a FITC dye at the N-terminus, which has a charge of −2 at the pH used in our experiments (pH 7.5). The ionic strength and temperature were also different (100 mM sodium phosphate buffer, *I* = 232 mM and 10°C). In these conditions the *K*_d_ is significantly lower than observed previously for similar length constructs (0.11 *μ*M).

In the NMR study, Sugase et al. were able to determine kinetic rate constants for a number of different residues in the complex. They fitted the data according to a simple three-state scheme (Eq. 2 below), involving formation of an intermediate with pKID partly bound to KIX:

2) $$\begin{array}{l} \text{pKID}+\text{KIX }\overset{k+}{\underset{k-}{⇄}}\text{I }\overset{k_{\text{IB}}}{\underset{k_{\text{BI}}}{⇄}}\text{pKID}:\text{KIX}\\ K_{\text{d}}=1.3\;\mu \text{M};k+=7.6\mu {\text{M}}^{-1}{\text{s}}^{-1};k-=7.6\;{\text{s}}^{-1}\text{;}\langle k_{\text{IB}}\rangle ∼1300\;{\text{s}}^{-1};\langle k_{\text{BI}}\rangle ∼500\;{\text{s}}^{-1}. \end{array}$$

We first examined our data to determine whether our results could be fitted to such a scheme. Compare our results:

$$K_{d}=0.11\;\mu \text{M;}\;k_{\text{ass,fast}}=7.3\mu {\text{M}}^{-1}{\text{s}}^{-1};k_{\text{diss}}=0.81\;{\text{s}}^{-1};k_{\text{ass,slow}}∼20\;{\text{s}}^{-1}.$$

The concentration-dependent on-rates (*k*+, *k*_ass,fast_) are essentially the same. The order of magnitude difference in *K*_d_ that we observe can be accounted for by a 10-fold difference in overall off rates, that is, in the stability of the complex. The overall off-rate is approximately *k*- in the Wright scheme (*k*- ≪ *k*_BI_), and is directly measured in our scheme.

We considered whether the single concentration independent rate we observe (*k*_ass,slow_ ∼20 s^−1^) could reflect exchange between the partly bound intermediate and the bound state that was observed by NMR, where the average *k*_IB_ + *k*_BI_ is reported to be ∼1800 s^−1^. Given the differences in conditions, in particular temperature, we cannot exclude this possibility. However, we note that our observed rate constant of 20 s^−1^ seems to be rather slow to be assigned to the same late folding and docking event.

There are two other possibilities:

**Figure:**
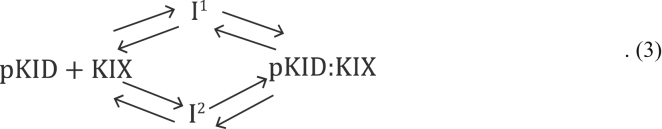

It is possible that NMR and stopped-flow experiments are detecting different intermediates. Indeed simulation studies (50) suggest that there might be two intermediates, which have different lifetimes and different populations. It may be that NMR would not detect the slow exchange between the proposed second intermediate and the bound state, just as we may be unable to detect the conversion of the NMR intermediate.

(4) $$\text{pKID}+\text{KIX }\overset{k+}{\underset{k-}{⇄}}\text{pKID}:\text{KIX}↔\text{pKID}*:\text{KIX}\text{.}$$

Finally, it is possible that the rate of exchange between *I* and the bound state are too fast to contribute to the kinetics of binding and unbinding (i.e., we cannot detect *I* in our kinetic experiments) and that our slow rate is detecting dynamics in the bound state(s) (pKID^∗^:KIX in Eq. 4). This could even be simply an artifact of the presence of the dye. Although we have ruled out photobleaching (data not shown) we note that we only observe this second rate using some extrinsic dyes, not others, and that we do not observe it when we use changes in intrinsic Tyr fluorescence to follow binding.

We do not have sufficient information to distinguish between these three possible schemes, and are thus unable to assign the second, low amplitude, unimolecular association phase. Importantly, however, just two kinetic rates appear to capture the overall kinetics of complex formation, since the *K*_d_ obtained from the two kinetic constants (*K*_d_ = *k*_ass,fast_/*k*_diss_) is the same as that obtained from the equilibrium experiment (0.11 *μ*M). We note that the *K*_d_ from kinetic experiments are 1.5–2.4 times lower than that from equilibrium for KID and KID-S133E, respectively; however, these values are determined with less certainty because of the significantly weaker binding (Fig. 1 *C*; Table 1). Moreover, the rate for the slow association phase is essentially unaffected by phosphorylation (or by the S133E mutation) (Fig. S3); we can therefore exclude this phase from further discussions here.

### Phosphorylation increases the lifetime of the complex

The main aim of our study was to investigate the mechanism whereby phosphorylation enhances the binding of KID to KIX. Structural and mutagenesis studies led to the suggestion that much of the increase in affinity upon phosphorylation can be ascribed to formation of specific electrostatic and hydrogen-bonding interactions in the bound state (46). However, the same authors also speculate that structural changes induced in the disordered peptide by phosphorylation influence affinity by increasing the population of a binding-competent species in the ensemble (30). Solt et al. (40) used molecular dynamics simulations to investigate the role of phosphorylation in KID:KIX recognition. They suggested that phosphorylation promotes formation of transient structures, similar to that of the bound conformation, through interactions with Arg 131. This is consistent with the increase in helicity that we observe in our experiments. However, they further proposed that since the transient conformations resemble the bound state, this kinked loop/turn where the serine becomes phosphorylated “acts as a primary contact site that establishes interactions with KIX first” (40). Our results are not consistent with this hypothesis, as we clearly show that phosphorylation does not increase the rate of association. Instead we find that the stability of the bound complex is almost entirely controlled by modulation of the dissociation rate. Wright and co-workers (29, 30), in their structural studies, demonstrated that pSer133 forms interactions both with Tyr 658 in KIX, but also with Arg 131 in pKID. Indeed in the accompanying article we show that truncation of R131 results in significant loss of stability of the complex (but no decrease in association rate) (51). Added to this may be a reduction in the entropic cost of folding brought about by the induced increase in helicity observed in MD simulations (42) and our CD experiments.

### S133E as a model for phosphorylation

Parker et al. (46) previously demonstrated that KID-S133E showed no affinity to KIX in vitro and did not support target gene activation in vivo. Our results now explain this observation. Here we show only a small increase in binding affinity of KID-S133E to KIX (over KID), still two orders of magnitude weaker than pKID (Fig. 2; Table 1). Moreover, the association kinetics is different; we observe a significant change in association rate (*k*_ass,fast_) for KID-S133E, whereas KID and pKID associate at essentially the same rate (Fig. 1 *D*; Table 1). It is perhaps surprising that the change in charged state upon phosphorylation, or upon mutation of Ser133 to Glu, does not induce an increase in association rate due to long-range electrostatic effects (34, 53). Also, specific and even off-pathway interactions can alter such electrostatics behavior (54). Our results make it clear that care must be taken when reporting results from phosphomimetics to represent the effect of phosphorylation in proteins and IDPs; a similar conclusion was drawn from simulations on the effect of phosphorylation in MDM2 (15).

## Conclusion

Even in the absence of phosphorylation, KID binds rapidly to its partner KIX, forming a short-lived complex. Phosphorylation has little effect on the rate of association, but allows formation of a complex with a significantly longer lifetime (half-life of ∼1 s for pKID vs. ∼10 ms for KID). We propose that modulating the lifetime of the complex by phosphorylation would allow regulation of recruitment of the transcriptional machinery to the CREB-response element sites. This mechanism could be biologically advantageous as it allows KID to act as a rapid switch for downstream signal transduction, allowing sufficient time for the transcription apparatus to be recruited without pKID being out-competed by numerous other IDPs that bind to KIX (36, 55).

## Authors Contributions

L.D., S.L.S., and J.C. designed research. L.D. performed research. L.D., S.L.S., and J.C. wrote the manuscript.

## Supporting Material

Document S1. Figs. S1–S3 and Table S1

Document S2. Article plus Supporting Material
