# Supplementary material for: Phosphorylation of the IDP KID Modulates Affinity for KIX by Increasing the Lifetime of the Complex
Source: Biophys J. 2017 Dec 19;113(12):2706–12. doi: 10.1016/j.bpj.2017.10.015 (PMC5770967; doi:10.1016/j.bpj.2017.10.015)
Supplement: Document S1. Figs. S1–S3 and Table S1 [file mmc1.pdf]

Biophysical Journal, Volume *113*

Supplemental Information

Phosphorylation of the IDP KID Modulates Affinity for KIX by Increasing the Lifetime of the Complex

Liza Dahal, Sarah L. Shammas, and Jane Clarke

Supplementary Table

Table S1. Fluorescence change and apparent rates for KIX association with each pKID version (determined at KIX concentration of 31μM)

pKID version*	Total amplitude	$k_{app,slow}$ (s^{-1})	$k_{app,fast}$ (s^{-1})
[a]Alexa488	0.40		169.3 ± 1.4
[a]Alexa546	1.87	11.3 ± 1.4	319 ± 2
[a]Alexa594	0.05		230 ± 40
[b]Tyrosine	0.30		82.3 ± 0.4
[c]FITC	0.25	23.1 ± 1.1	181.4 ± 1.4

Observed association kinetic rates and total change in fluorescence intensity of different N-terminally labelled pKID upon association with KIX (ratio 1:10). pKID was N-terminally labelled with different extrinsic dyes and the overall change in amplitude and the observed association rates are compared with unlabelled (intrinsic tyrosine fluorescence). Different extrinsic fluorescence dyes give a different overall change in amplitude. Two rates ($k_{app,fast}$ and $k_{app,slow}$) are observed for Alexa546 and FITC labelled pKID association with KIX and a single rate ($k_{app,fast}$) is observed for Alexa488, Alexa594 and pKID with intrinsic tyrosine fluorescence.

*Peptide sequences for the different pKID versions are shown below

[a] Alexa-CVDSVTDSQKRREILSRRPpSYRKILNDLSSDAP

[b] DSVTDSQKRREILSRRPpSYRKILNDLSSDAP

[c] FITC-Ahx-DSVTDSQKRREILSRRPpSYRKILNDLSSDAP

Supplementary Figures

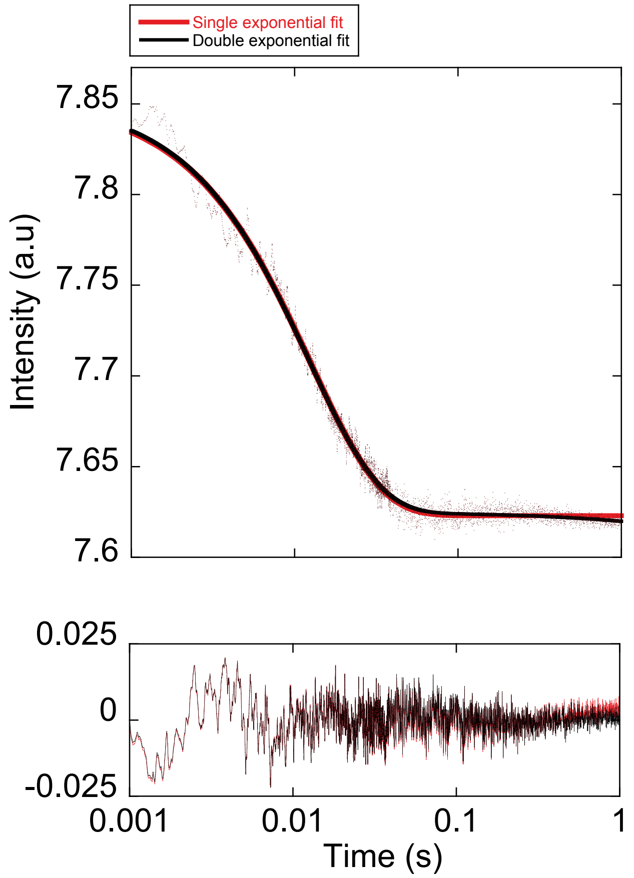

Figure S1. Change in tyrosine fluorescence of pKID on mixing with KIX (ratio 1:10). An average of approximately 90-100 kinetic traces (collected on a stopped-flow fluorimeter) is shown. The kinetic data fits equally well to both single and double exponential. Only a single kinetic phase is detected with tyrosine fluorescence.

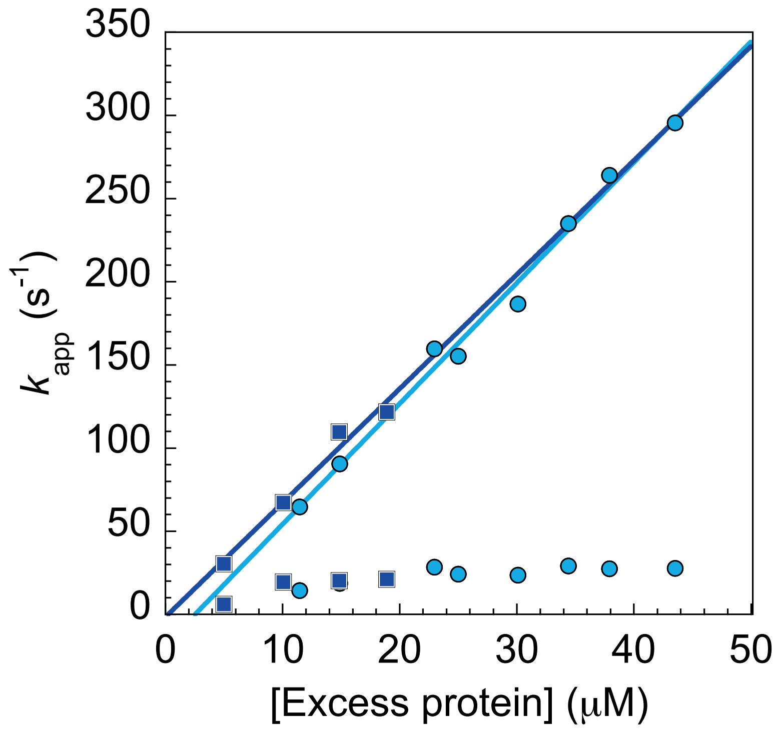

Figure S2. FITC-pKID binding to KIX, under pseudo-first order and reverse pseudo-first order conditions. Two phases, one concentration dependent (fitted to a straight line) and another concentration independent are detected. Observed association rates from experiments with KIX in excess (light blue spheres) and pKID in excess (dark blue squares) are plotted against different concentrations of protein in excess.

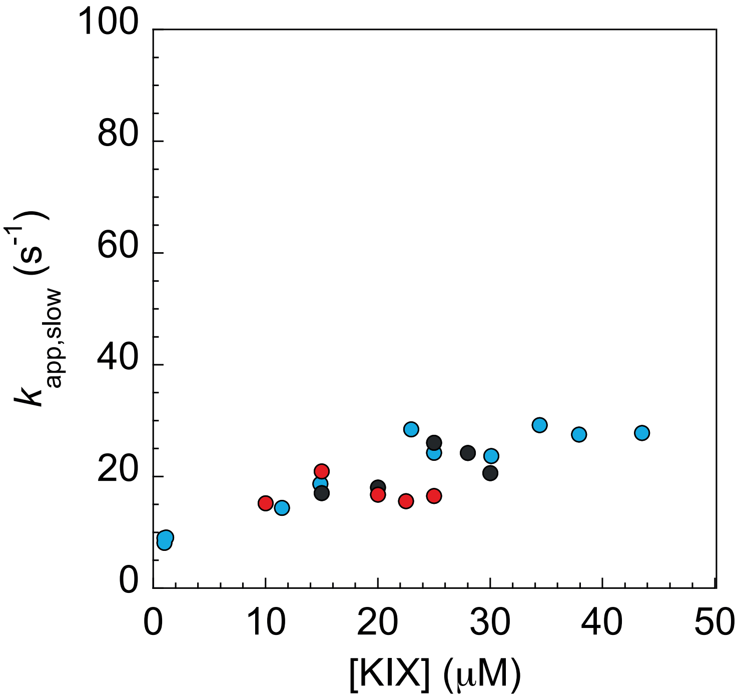

Figure S3. Slow concentration independent rates are observed for pKID (blue), KID (black) and S133E (red) association with KIX.
